# HnRNP-L mediates bladder cancer progression by inhibiting apoptotic signaling and enhancing MAPK signaling pathways

**DOI:** 10.18632/oncotarget.14600

**Published:** 2017-01-11

**Authors:** Daojun Lv, Huayan Wu, Rongwei Xing, Fangpeng Shu, Bin Lei, Chengyong Lei, Xumin Zhou, Bo Wan, Yu Yang, Liren Zhong, Xiangming Mao, Yaguang Zou

**Affiliations:** ^1^ Department of Urology, Nanfang Hospital, Southern Medical University, Guangzhou 510515, P. R. China; ^2^ Department of Urology, Peking University Shenzhen Hospital, Shenzhen PKU-HKUST Medical Center, Shenzhen, Guangdong Province, 518036, China; ^3^ Department of Stomatology, Nanfang Hospital, Southern Medical University, Guangzhou 510515, P. R. China; ^4^ Department of Urology, Affiliated Weihai Second Municipal Hospital of Qingdao University, Weihai 264200, P. R. China

**Keywords:** bladder cancer, heterogeneous nuclear ribonucleoprotein L, epithelial-mesenchymal transition, intrinsic apoptosis, MAPK

## Abstract

Heterogeneous nuclear ribonucleoprotein L (hnRNP-L) is a promoter of various kinds of cancers, but its actions in bladder cancer (BC) are unclear. In this study, we investigated the function and the underlying mechanism of hnRNP-L in bladder carcinogenesis. Our results demonstrated that enhanced hnRNP-L expression in BC tissues was associated with poor overall survival of BC patients. Depletion of hnRNP-L significantly suppressed cell proliferation *in vitro* and inhibited xenograft tumor growth *in vivo*. Furthermore, downregulation of hnRNP-L resulted in G1-phase cell cycle arrest and enhanced apoptosis accompanied by inhibition of EMT and cell migration. All these cellular changes were reversed by ectopic expression of hnRNP-L. Deletion of hnRNP-L resulted in decreased expression of Bcl-2, enhanced expression of caspases-3, -6 and -9 and inhibition of the MAPK signaling pathway. These findings demonstrate that hnRNP-L contributes to poor prognosis and tumor progression of BC by inhibiting the intrinsic apoptotic signaling and enhancing MAPK signaling pathways.

## INTRODUCTION

Bladder cancer (BC) is one of the most common malignant neoplasms in urological system and considered as the fourth most prevalent neoplasm in males [1]. Its incidence has been increased dramatically in the past decades [2]. Despite the greatly improvement in surgical techniques and adjuvant therapies, recurrence and progression of local tumor, distant metastases and poor prognosis are still the challenges [3, 4]. Tumorigenesis of bladder cancer is a multi-gene, multi-factor and multistep process in company with the environmental and genetic factors. Nevertheless, the complicated pathways during tumor formation remain unclear. Thus, it is urgent to identify novel biomarkers and therapeutic targets for the prognostic prediction and treatment of bladder cancer.

Heterogeneous nuclear ribonucleoprotein L (hnRNP-L) is a component of the hnRNP family that is typically interacted with hnRNP complexes to regulate different pre-mRNA and mature mRNA transcription [5, 6]. Several complicated mechanisms of hnRNP-L have been clarified, such as splicing regulation [7], transport of intronless mRNAs [8], IRES-mediated translation [9] and regulation of RNA stabilization [10]. Recent studies reported that hnRNP-L upregulation played key roles in regulating cell proliferation, apoptosis, metastasis, invasion and angiogenesis in a few types of malignances, including non-small cell lung cancer [11], colorectal cancer [12] and breast cancer [13]. Moreover, hnRNP-L has been reported to be overexpressed in hepatocellular carcinoma (HCC) and associated with the increased tumor size and reduced survival rate of HCC patients and knocked down hnRNP-L significantly inhibited cell growth, migration and invasion in vitro [14]. However, the correlation between hnRNP-L and bladder cancer has not been characterized.

Mitogen-activated protein kinase (MAPK) family members are crucial intracellular signaling molecules in various cellular processes, including proliferation, migration, invasion and apoptosis [15]. Conventional MAPKs mainly divided into four major groups: the extracellular signal-regulated kinase 1 and 2 (ERK1/2), the c-Jun N-terminal kinases 1–3 (JNK1-3), the p38 isoforms (p38α, β, γ, and δ) and ERK5, which are involved in tumor progression and metastasis [16]. Moreover, cumulative evidence indicates that the epithelial-to-mesenchymal transition (EMT) is regarded as an imperative process in tumor metastasis, which involved in cell migration, invasion, apoptosis [17]. Both MAPK and EMT signal pathways play vital characters in the initiation and progression of bladder cancer [18, 19]. However, it remains undefined whether hnRNP-L can regulate MAPK and EMT signal pathways in bladder cancer.

So far, the role of hnRNP-L in bladder cancer tumorigenesis has not been well illuminated. In the present study, we measured the endogenous expression and clinical significance of hnRNP-L in bladder cancer. Additionally, the effect of abnormalities hnRNP-L expression on cellular biology and the underlying mechanism was also explored.

## RESULTS

### HnRNP-L is up-regulated in human bladder cancer tissues

**Figure 1:**
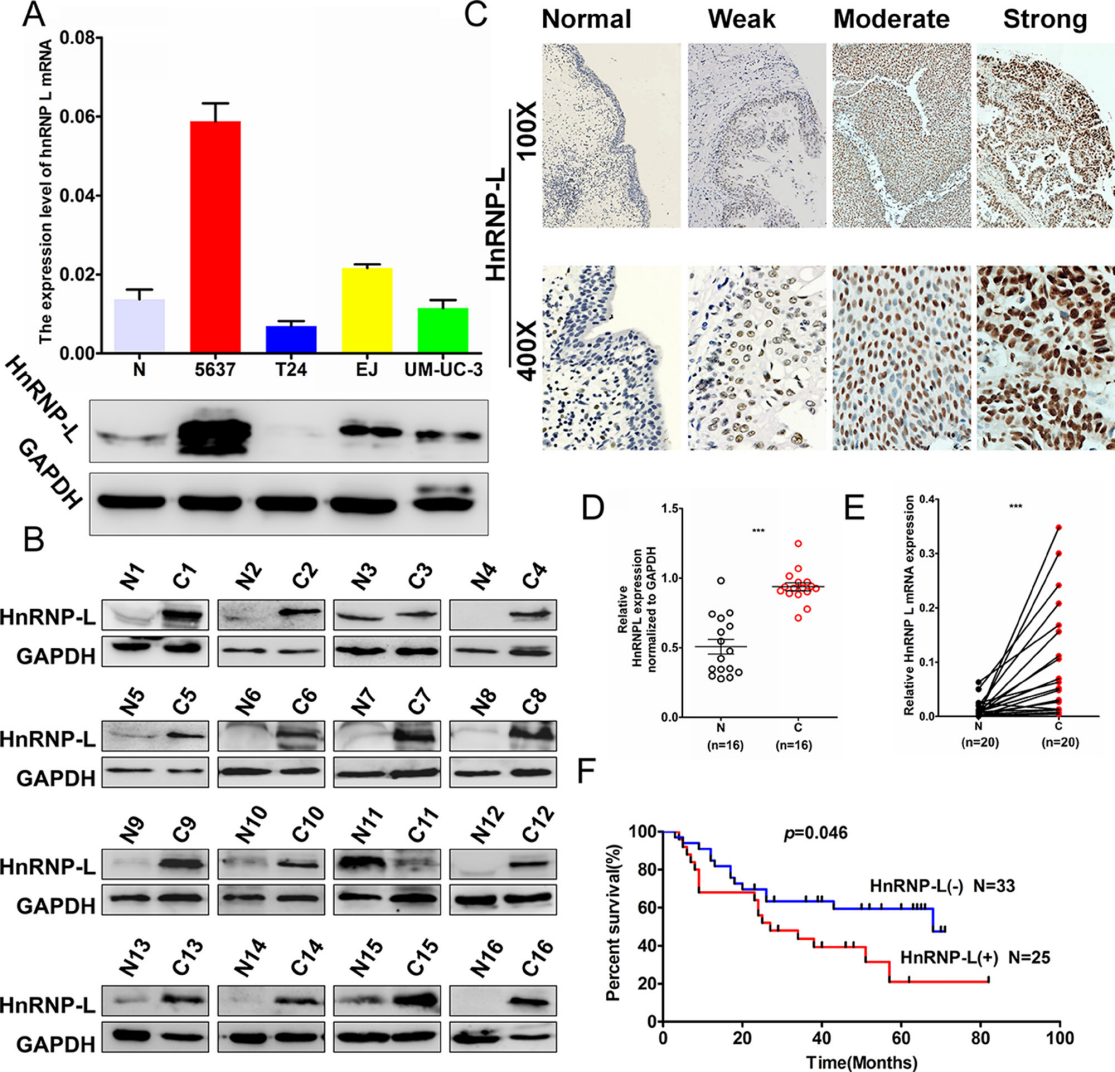
HnRNP-L expression in bladder cancer cell lines and the patients’ tissues **(A)** Western blotting and real-time qPCR analysis of hnRNP-L protein and mRNA expression in bladder cancer cell lines, N represented the normal human urinary tract epithelial cells SV-HUC-1; **(B)** HnRNP-L protein expression in bladder normal (N) and matched cancer tissues (C) were examined by Western blotting (n = 16); **(C)** Representative images of hnRNP-L protein immunochemistry in normal bladder tissues and bladder cancer tissues. Magnification: ×100, ×400. C1. Normal bladder tissues; C2. weak positive staining in bladder cancer tissue; C3. moderate positive staining in bladder cancer tissue; C4. Strong positive staining in bladder cancer tissue; **(D)** HnRNP-L mRNA in bladder cancer (C) and adjacent normal bladder tissues (N) were analysed by real-time qPCR (n = 20, p < 0.001). The levels of hnRNP-L protein and mRNA were shown as mean ± SD; **(E)** The relative levels of hnRNP-L protein were quantified by using image J and normalized to internal control GAPDH (n = 16, p < 0.001); **(F)**. The Kaplan-Meier overall survival curve of bladder cancer patients (n = 58) according to hnRNP-L protein expression (p = 0.046, by the log-rank test).

To investigate the expression profile of hnRNP-L in human bladder cancer, we detected hnRNP-L expression in four human bladder cancer cell lines (i. e. UM-UC-3, EJ, T24 and 5637) and found that the mRNA and protein levels of hnRNP-L was relatively higher in 5637 and EJ cells to T24 and UM-UC-3 cells (Figure 1A). To further confirm the correlation of hnRNP-L expression with tumor progression in bladder cancer. We performed Western blotting and qRT-PCR assays in human tissue samples and showed that hnRNP-L protein expression was markedly higher in bladder cancer tissues than those in adjacent normal bladder tissues (Figure 1B and Figure 1D, n = 16, *p* < 0.001). In addition, the mRNA level of hnRNP-L was also dramatically increased in bladder cancer tissues than those in matched nontumorous tissues (Figure 1E, n = 20, *p* < 0.001). Then, hnRNP-L expression was detected in tissue microarray (TMA) containing 155 cases of archived paraffin-embedded bladder cancer specimens by immunohistochemical staining (IHC). Results demonstrated that hnRNP-L was increased significantly in most of bladder cancer tissues. Whereas was seldom detected in adjacent nontumorous tissues (Figure 1C and Table 1) (*p* < 0.001). As showed in Table 2, up-regulation of hnRNP-L was significantly associated with pT stage (*p* = 0.025), pathology grade (*p* < 0.001), clinical stage (*p* = 0.003) and age (*p* = 0.001). However, no significant correlation was found between hnRNP-L protein expression with gender. Kaplan–Meier analysis for 58 patients with follow-up data suggested that patients with higher levels of hnRNP-L presented significantly lower overall survival rates than those with low levels of hnRNP-L expression (Figure 1F, Log rank, *p* = 0.046). Furthermore, the multivariate analysis of the Cox regression model, hnRNP-L expression (*p* = 0.002, HR = 5.038) and tumor clinical stage (*p* = 0.018, HR = 8.456) was confirmed to be independent prognosis factors for bladder cancer patients (Table 3). These results suggested that hnRNP-L may be a probable independent predictor in patients with bladder cancer.

**Table 1:** The expression of HnRNP-L in normal bladder tissues and in a series of bladder cancer tissues

Type	*N*	HnRNP-L protein expression		*p* value
		Positive (%)	Negative (%)	
Normal bladder	46	10.9 (5)	89.1 (41)	< 0.001
Bladder cancer	155	77.4 (120)	22.6 (35)	

Values are *n* (%). A significant increasing frequency of positive expression of HnRNP-L was detected in bladder cancer compared to adjacent normal bladder tissue (*P* < 0.001, *χ*^2^-test).

**Table 2:** The correlation between HnRNP-L and clinicalpathologic characteristics was analyzed in bladder cancer by IHC (*n* = 155)

Clinicalpathologic characteristics	*N*	HnRNP-L		*p* value^b^
		Positive (%)	Negative (%)	
Age (years)				
< = 63^a^	74	66 (89.2)	8 (10.8)	0.001
> 63	81	54 (67.7)	27 (33.3)	
Gender				
Male	127	99 (74.0)	28 (26.0)	0.735
Female	28	21 (75.0)	7 (25.0)	
pT status				
Tis-T1	15	6 (40.0)	9 (60.0)	0.025
T2	90	77 (85.6)	13 (14.4)	
T3–T4	48	34 (70.8)	14 (29.2)	
Pathology grade				
I–II	99	87 (87.9)	12 (12.1)	0.000
III	53	30 (56.6)	23 (43.4)	
Clinical stage				
I	9	6 (66.7)	3 (33.3)	0.003
II	99	89 (89.9)	10 (10.1)	
III	32	21 (65.6)	14 (43.8)	

^a^ : mean age. ^b^: *p* value is from *χ*^2^-test.

**Table 3:** Univariate and multivariate analysis of different prognostic parameters in patients with bladder cancer by Cox regression analysis

Covariates	Univariate analysis		Multivariate analysis	
	*p* value	HR (95% CI)	*p* value	HR (95% CI)
HnRNP-L	0.052	2.030 (0.993–4.149)	0.002	5.038 (1.783–14.238)
Gender	0.380	1.495 (0.609–3.670)	0.133	2.600 (0.748–9.037)
Age	0.691	0.993 (0.958–1.029)	0.571	0.988 (0.946–1.031)
Clinical stage	0.025	2.075 (1.096–3.928)	0.018	8.456 (1.434–49.861)
pT stage	0.064	1.575 (0.975–2.547)	0.093	0.210 (0.034–1.295)
Pathology grade	0.286	1.532 (0.699–3.354)	0.986	1.000 (0.382–2.666)

Abbreviations: CI, confidence interval; HR: Hazard Ratio.

### HnRNP-L accelerates cell cycle progression and proliferation of bladder cancer cell lines

**Figure 2:**
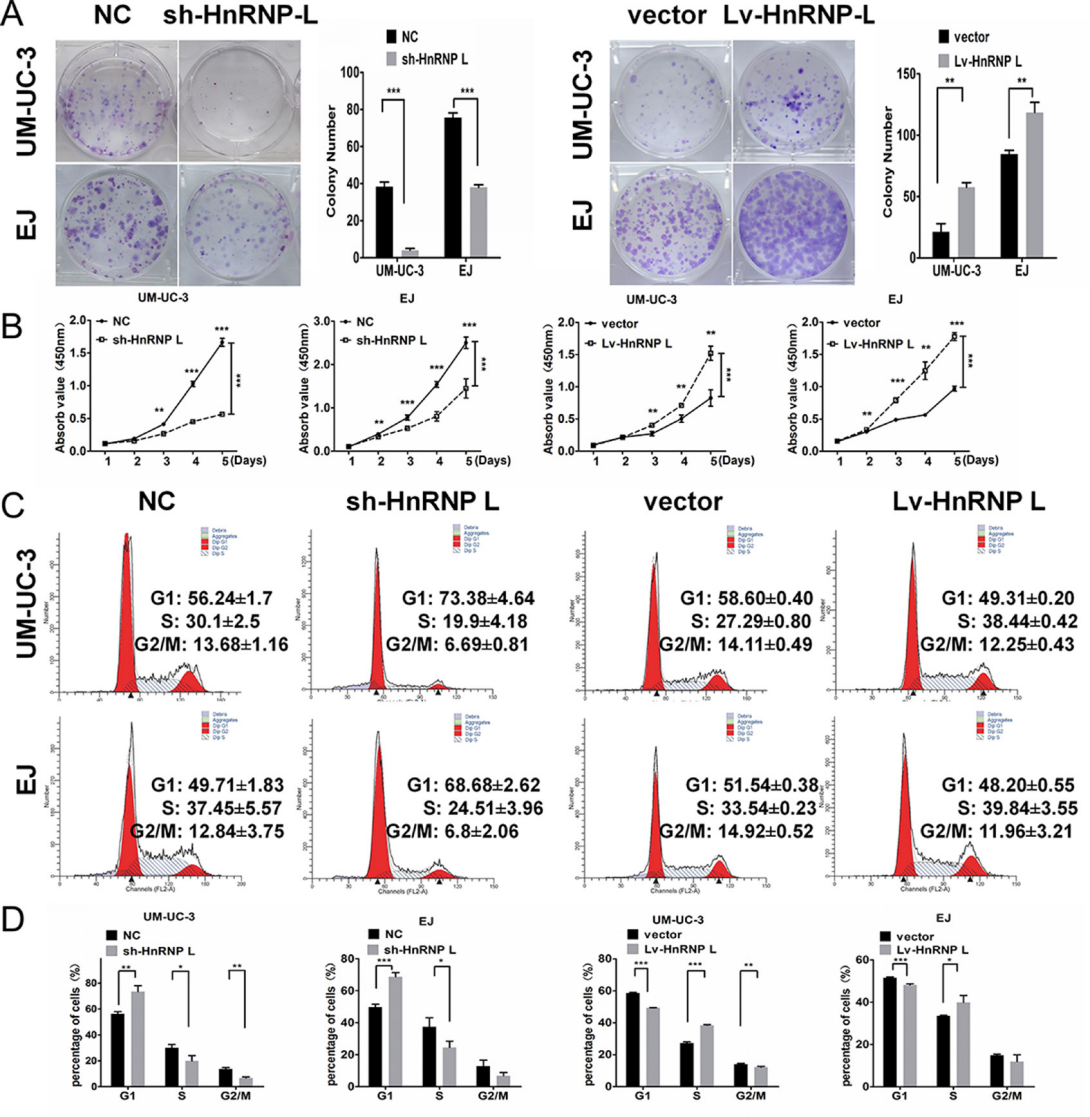
HnRNP-L stimulates proliferation and accelerates the cell cycle of UM-UC-3 and EJ cell lines **(A)** When cultured in the plate colony formation assay, colony formation by hnRNP-L transfected cells was obviously enhanced, and decreased in sh-hnRNP-L transfected cells; **(B)** In the CCK-8 assay, cell viability was increased by hnRNP-L and decreased by sh-hnRNP-L; **(C)** Flow cytometry revealed that sh-hnRNP-L transfected cells were arrested in G0/G1 phase; Conversely, hnRNP-L transfected cells were accelerated into the S phase; **(D)** Vertical bars presented the statistical analysis of the cell cycle results. All the assays were each performed three times independently. The data are shown as mean ± SD *p < 0.05; **p < 0.01; ***p < 0.001.

To investigate the effect of hnRNP-L on cell proliferation, we down-regulated and up-regulated the expression of hnRNP-L in UM-UC-3 and EJ cells by lentivirus vectors. Subsequently, we validated the infection efficiencies using Western blotting and showed that the expression of hnRNP-L protein was significantly increased in the hnRNP-L overexpression group and obviously decreased in hnRNP-L knockdown group in the transfected bladder cancer cells (Supplementary Figures 1–2). In both cell lines, proliferation was revealed to be accelerated by ectopic-hnRNP-L and suppressed by sh-hnRNP-L. As showed in Figure 2A, clone formation assay revealed that the number and size of colonies were remarkably reduced in sh-hnRNP-L cells, while were remarkablely increased in hnRNP-L overexpression cells. CCK-8 assay revealed that cell proliferation was significantly promoted by hnRNP-L upregulation, but inhibited by sh-hnRNP-L (Figure 2B).

To validate whether the proliferation suppression upon sh-hnRNP-L is involved in the cell cycle profile alterations, flow cytometry was used to quantify the cellular DNA content. Results showed that hnRNP-L depletion induced cycle arrest in G1 phase. While hnRNP-L overexpression accelerated growth progression into S phase (Figure 2C–2D).

**Figure 3:**
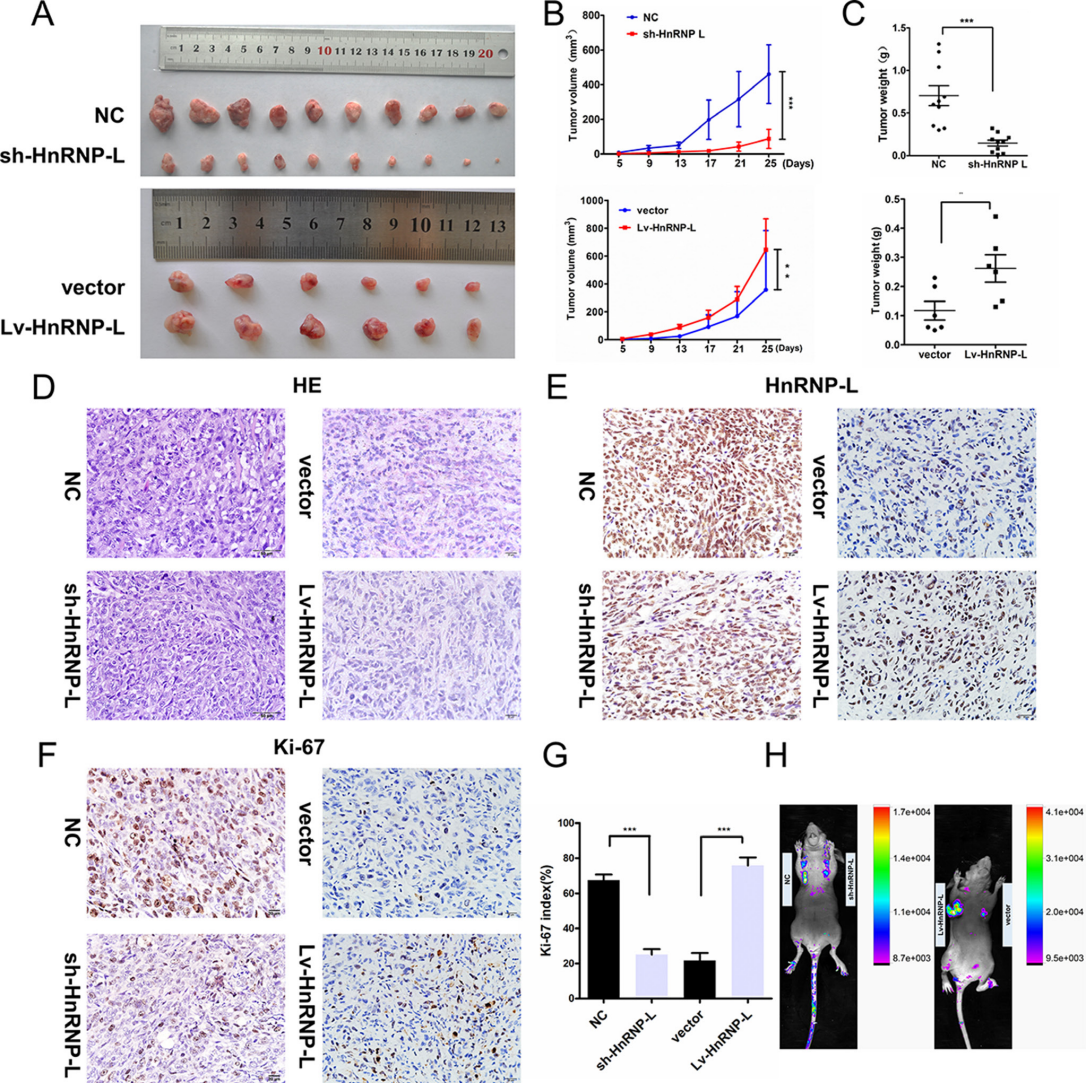
HnRNP-L accelerates tumor growth in vivo **(A)** Gross observation of xenograft tumor size in NOD/SCID mice; **(B)** hnRNP-L overexpression promotes tumour growth, while silencing of hnRNP-L inhibited the tumor growth; **(C)** Histological analysis of tumor weight (vs. Control). **(D)** H&E-stained paraffin-embedded sections obtained from xenografts. **(E)** IHC staining for hnRNP-L of in xenograft tumors. **(F–G)** IHC staining for Ki-67 in xenograft tumors and the histogram exhibited the Ki-67 index (%). **(H)** The infection efficacy of lentivirus with GFP in vivo. Original magnification (×400). The data are shown as mean ± SD *p < 0.05; **p < 0.01; ***p < 0.001.

To confirm the impact of hnRNP-L depletion on tumor growth *in vivo*, we established a subcutaneous xenograft tumor model in athymic nude mice by injecting EJ-sh-hnRNPL cells and control EJ-NC cells. As expected, the cells with hnRNP-L depletion resulted in significantly slower-growing xenografts as compared to cells with negative controls. Whereas, the cell with hnRNP-L overexpression in UM-UC-3 showed faster-growing xenografts than the control cells (*p* < 0.05, Figure 3A–3C). Haematoxylin and eosin (H&E) staining revealed that the histopathological features of the tumor tissues (Figure 3D) and Immunological Histological Chemistry IHC was performed to determine the expression of hnRNP-L in xenograft tumors (Figure 3E). The immunohistochemistry (IHC) of the tumor tissues from xenografts demonstrated that the expression of Ki-67 proliferation antigen was significantly weaker in xenografts of sh-hnRNP-L cells and remarkablely stronger in xenografts of upregulation hnRNP-L (Figure 3F–3G). The infection efficacy of lentivirus with GFP *in vivo* was confirmed by animal *in vivo* imaging instrument (Figure 3H).

Taken together, these results provided evidence that hnRNP-L presented tumor-promoting property to promote cell proliferation and accelerate cell cycle progression from G1 to S phase in bladder cancer.

### HnRNP-L depletion results in the intrinsic apoptosis of bladder cancer

**Figure 4:**
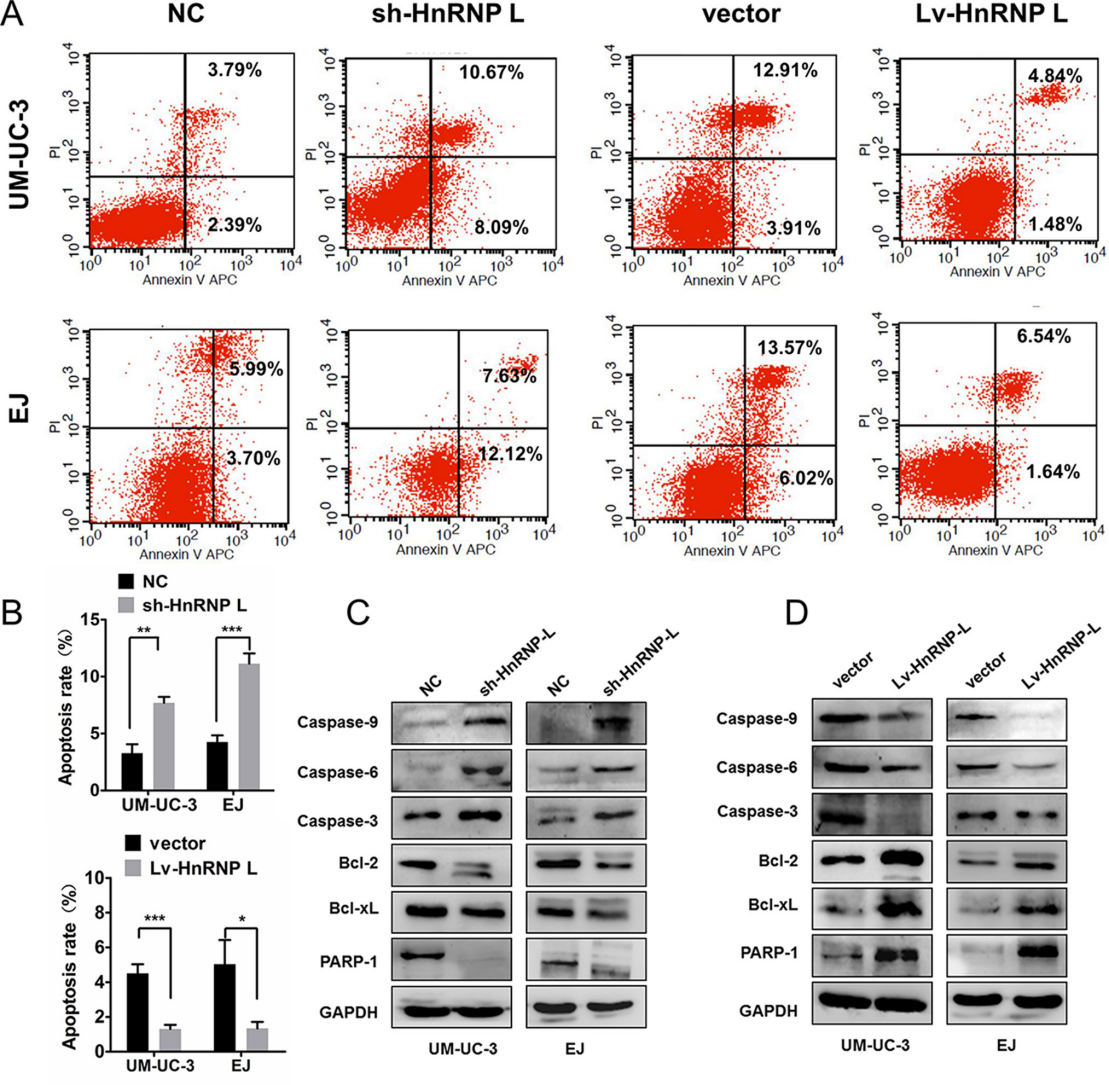
HnRNP-L expression affected the apoptosis in UM-UC-3 and EJ cells **(A)** HnRNP-L knockdown increased the rate of apoptosis by flow cytometry; Whereas hnRNP-L overexpression decreased the rate of apoptosis by flow cytometry; **(B)** Statistics analysis of the apoptosis rate in each group (n = 3, Student's t-test, p < 0.05). Means ± SD were shown, *p < 0.05; **p < 0.01; ***p < 0.001; **(C)** Western blot shown that hnRNP-L knockdown increased the expression caspase-9, caspase-6, caspase-3 and Bcl2L1, whereas the expression of Bcl-2 and PARP-1 was decreased; **(D)** HnRNP-L overexpression reversed all the changes on the apoptosis pathway. GAPDH served as loading control.

We further examined the influence of hnRNP-L expression on cell apoptosis by flow cytometry. Apoptosis rates in UM-UC-3 and EJ cells with hnRNP-L down-regulation were significantly higher than those negative controls (Figure 4A–4B). On the contrary, apoptotic resistance was presented in ectopic-hnRNP-L bladder cancer cells (Figure 4A–4B).

As far as we known, the caspases, Bcl-2, Bcl-xL and PARP were related to cell apoptosis in bladder cancer [20, 21]. Thus, we measured the activation of Bcl-2/caspases pathway. Results exhibited that hnRNP-L knockdown activated the caspase-3, caspase-6, caspase-9, but inhibited the activation of Bcl-2, Bcl-xL and PARP in UM-UC-3 and EJ cells (Figure 4C). However, hnRNP-L overexpression received an inverse effect (Figure 4D). Therefore, these results indicated that hnRNP-L overexpression suppressed intrinsic apoptosis by blocking Bcl-2, caspases-3, -6 and -9 pathways.

### HnRNP-L promotes the migration of bladder cancer cell by EMT

**Figure 5:**
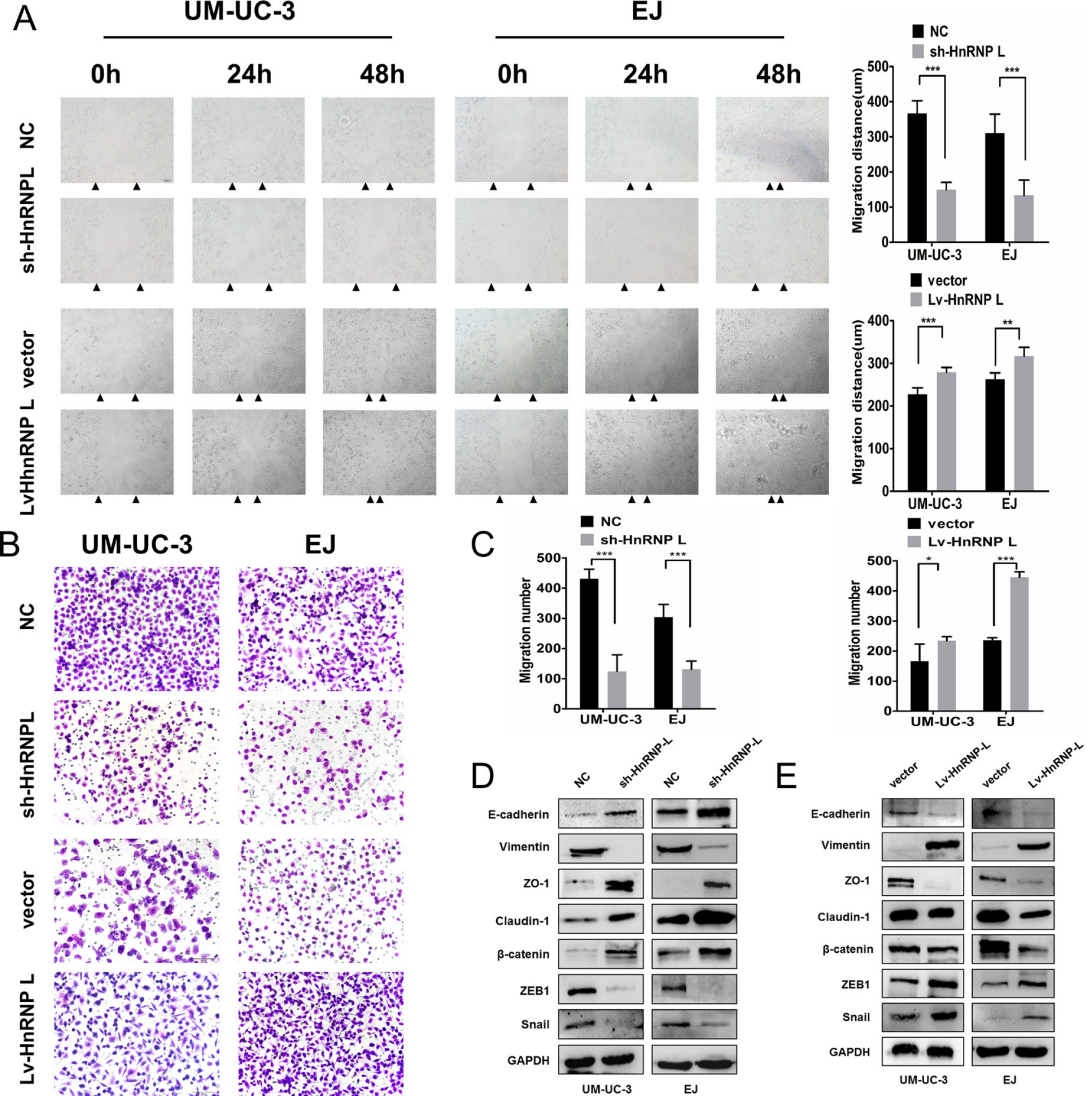
HnRNP-L overexpression enhances migration in UM-UC-3 and EJ cells **(A)** Migration of cells was assessed by the wound closure assay. After 48 h, the wound was nearly closed by hnRNP-L transfected cells and was wider with sh-hnRNP-L transfected cells. Vertical bars indicated the mean distance of cell migration as compared with the negative control; **(B)** In the transwell assay, migration was quantified by cells migrating through the bottom chamber. The cell numbers were significantly increased in hnRNP-L transfected cells and were decreased in sh-hnRNP-L transfected cells; **(C)** Statistics analysis of the mean migration cell numbers as compared with the negative control. All experiments were performed three times independently, *p < 0.05; **p < 0.01; ***p < 0.001; **(D)** In both cell lines. EMT markers were modulated by hnRNP-L expression. Epithelial markers (E-cadherin and β-catenin) and the tight junction proteins (ZO-1 and caludin-1) were promoted when hnRNP-L knockdown, whereas mesenchymal markers (Vimentin) and the repressor of E-cadherin (snail, ZEB1) were down-regulated; **(E)** While opposite results showed in hnRNP-L overexpressed cells. GAPDH served as loading control.

Then, we evaluated the effect of hnRNP-L expression on cell migration. Wound healing assay revealed that hnRNP-L downregulation significantly inhibited cell migration. Whereas hnRNP-L overexpression dramatically enhanced cell migration in UM-UC-3 and EJ cells (Figure 5A). Transwell assay showed that the number of cells that migrated through the chamber was significantly greater in hnRNP-L overexpression group, and significantly decreased in hnRNP-L knockdown group than those in control groups (Figure 5B–5C).

It has been widely known that Epithelial-Mesenchymal Transition (EMT) is closely correlated with cell migration [22]. Thus, we investigated whether hnRNP-L affected cell migration by EMT in bladder cancer. Results revealed that hnRNP-L down-regulation inhibited the expression of mesenchymal markers (Vimentin) and the repressor of E-cadherin (snail and ZEB1), but increased the expression of E-cadherin, β-catenin and the tight junction proteins (ZO-1 and caludin-1) in UM-UC-3 and EJ cells (Figure 5D). In contrast, hnRNP-L overexpression unregulated the expression of Vimentin, ZEB1 and snail while downregulated the expression of E-cadherin, β-catenin, ZO-1 and caludin-1 (Figure 5E). These data demonstrated that hnRNP-L expression promoted cell migration by EMT, which might exert an important role in bladder cancer progression.

### HnRNP-L knockdown suppressed the activation of MAPK pathways

**Figure 6:**
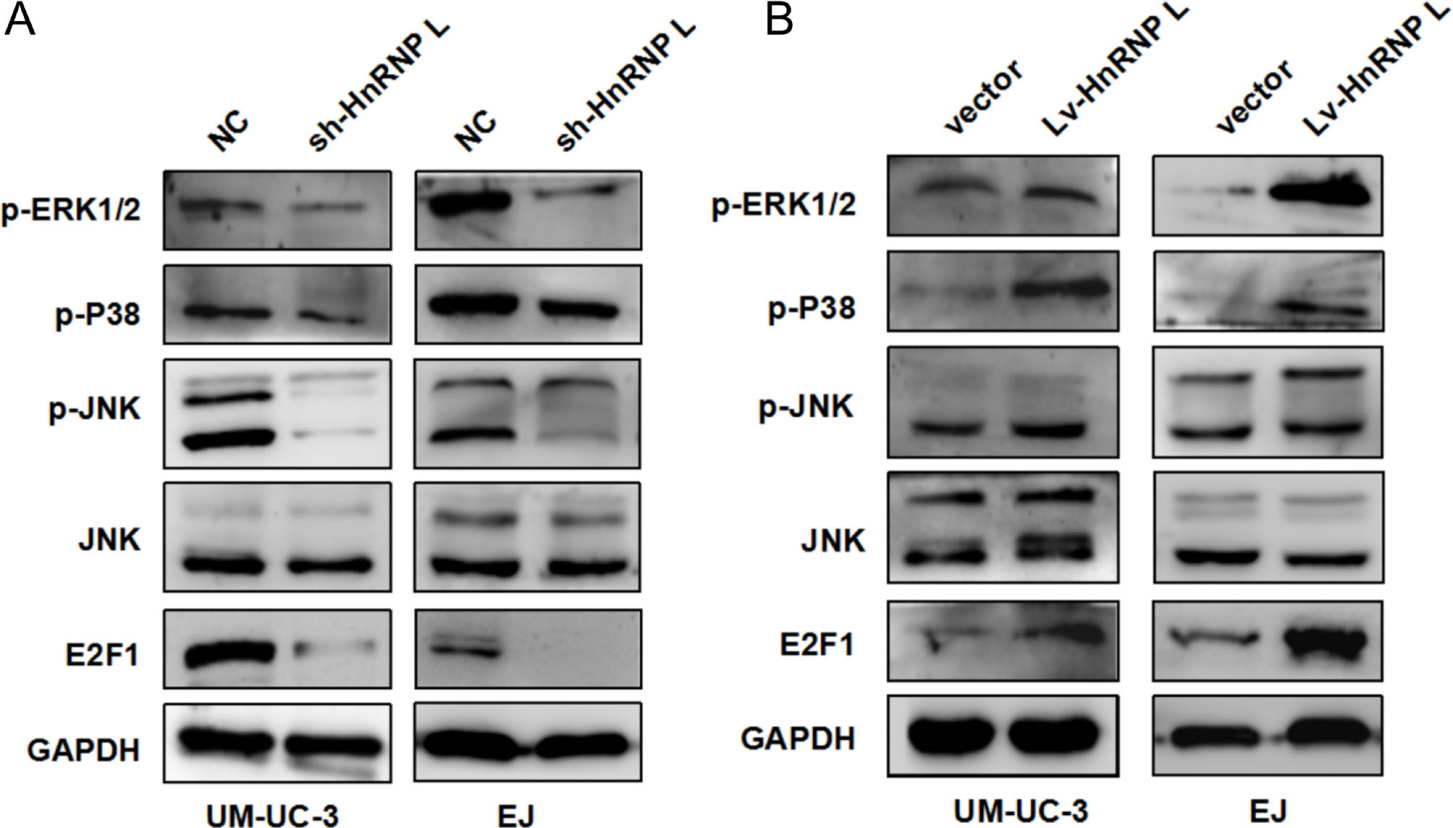
HnRNP-L expression affected the activation of MAPK signaling pathways **(A)** Levels of p-ERK1/2, p-JNK, p-P38 JNK and E2F1 were determined by Western blotting; **(B)** Effect of hnRNP-L overexpression on MAPK pathway and E2F1. GAPDH served as loading control.

MAPKs mainly consisting of ERK1/2, JNK and p38, play critical roles in cell apoptosis, proliferation and migration. Simultaneously recent literature dictated that ERK/MAPK was regulated by the transcription factor E2F1, which medicated the G1/S checkpoint [23]. Thus, we examined the activation of the MAPK pathway and E2F1 to further uncover the underlying mechanisms of hnRNP-L in bladder cancer. Western blotting results showed that the expression of E2F1 and phosphorylation of ERK1/2, p38 and JNK was reduced in UM-UC-3 and EJ cells with hnRNP-L knockdown (Figure 6A). On the contrary, the results were reversed in the cells with hnRNP-L overexpression (Figure 6B). These results implied that the activation of MAPKs and E2F1 regulated by hnRNP-L might be also responsible for cell proliferation, apoptosis and migration.

## DISCUSSION

The hnRNP-L belongs to the family of hnRNPs that regulates the formation, processing and stability of the mRNAs. Though mutations in hnRNP-L are as associated with pancreatic cancer [24], oral squamous cell carcinoma cells (OSCC) [25] and breast cancer [13], its role in bladder cancer is uncertain. Therefore, our study confirmed that enhanced hnRNP-L in cancerous bladder tissues in comparison to normal bladder epithelium correlated with lower survival rates. Furthermore, our study confirmed that hnRNP-L was an independent prognostic factor for overall survival of bladder cancer patients. This was consistent with similar observations regarding hnRNP-L in Hepatitis B virus- related hepatocellular carcinoma[14].

Previously, hnRNP-L has been implicated in tumorigenesis. Goehe and colleagues showed that depletion of hnRNP-L suppressed the tumorigenic capacity in NSCLC through the post-transcriptional processing of caspase-9 pre-mRNA [11]. Gaudreau and others showed that silencing of hnRNP-L affected T-cell activation [26]. This study demonstrated that knockdown of hnRNP-L in bladder cancer cells significantly inhibited cell proliferation, migration and EMT and induced cell apoptosis as well as G1-S phase transition arrest. These data suggested that hnRNP-L played a critical role in tumor initiation and progression of bladder cancer.

Aberrant apoptosis can modulate carcinogenesis and lead to the limitless proliferation of tumor due to dysregulated downstream effectors. Members of the caspase families and the Bcl-2 family of proteins that are central regulators of the intrinsic apoptosis pathway also participate in the apoptosis of bladder cancer cells [27, 28]. The members of the caspase families are crucial regulators of apoptosis by recruiting and activating initiator caspases that result in programmed cell death [29]. The binding of hnRNP-L to the ‘CA’ repeats in the Bcl-2 mRNA results in its decay and therefore can affect the stability of the mitochondrial membrane that is maintained by the anti-apoptotic proteins, Bcl-2 and Bcl-xL [30, 31]. Furthermore, hnRNP-L was shown to be critical for the survival and functional integrity of hematopoietic stem cells by restricting the activation of caspase-dependent death receptor pathways [32]. Our study demonstrated that hnRNP-L down-regulation resulted in the apoptosis of bladder cancer cells due to activation of apoptosis inducing factors, caspase-3/6/9 and suppression of apoptosis inhibitory factors, Bcl-2, Bcl-xL and PARP1. These results implied that hnRNP-L up-regulation in bladder cancer promoted anti-apoptotic function and cell proliferation by suppressing the intrinsic caspase-dependent apoptosis cascade.

EMT ( Epithelial-Mesenchymal Transition) plays a pivotal role in the process of tumor progression and metastasis of solid tumors [33]. Recently, hnRNP-L was shown to bind the VEGFA mRNA and inhibit miRNA activity, thereby mediating tumor metastasis [34, 35]. Therefore, we tested if hnRNP-L regulated EMT in bladder cancer metastasis and found that hnRNP-L depletion maintained the epithelial phenotype and inhibited mesenchymal transition, whereas overexpression of hnRNP-L promoted EMT. These results showed that hnRNP-L promoted cell migration and enhanced metastasis of bladder cancer.

Regarding signaling pathways involved in cancer, the mitogen-activated protein kinases (MAPKs) that comprise of the ERK1/2, JNK and p38 MAPK which play key roles in regulating multiple cellular processes like cell proliferation, motility and survival [15, 36]. The abnormal activation of ERK1/2 pathway facilitates tumorigenesis, whereas, the stress-induced activation of JNK and p38MAPK controls the balance of cell autophagy and apoptosis [37, 38]. In this study, we observed that hnRNP-L upregulated p-ERK, p-p38 and p-JNK suggesting that hnRNP-L regulated cell fate in bladder cancer cells by promoting the MAPK (ERK/JNK/p38MAPK) signaling pathway.

In summary, our findings show evidence that hnRNP-L can be a critical prognostic biomarker for bladder cancer and targeted therapy. However, clinical studies are necessary to validate the utility of hnRNP-L in bladder cancer prognosis and therapy.

## MATERIALS AND METHODS

### Patients and clinical pathology data

A total of 155 patients (mean age 63.3 years, range 25–85) diagnosed with primary bladder cancer who underwent operation at the Department of Urology of the Peking University Shenzhen Hospital (Shenzhen, China) and Nanfang hospital (Guangzhou, China) between January 2007 and February 2011 were enrolled in our study. Parts of tissues were fixed in 10% formalin for routine immunohistochemical assays and remainder tissues were stocked at –80°C for further processing. The follow up of participants (*n* = 58) were gotten through phone calls until death or the cut-off date of December 29, 2013. The mean follow-up time was 32 months (from 3.0 to 82.0 months). All the deaths were ascribed to bladder cancer. Patients’ characteristics were retrospectively collected from the review of medical records and detailed in Table 2. Pathological TNM staging was reassessed in accordance with the American Joint Committee on Cancer (AJCC). Histological grade was assigned basing on the World Health Organisation (WHO) classification (2004). All patients arranged their written informed consent to participate in this study and the study protocol was authorized by the Ethics Committee of Peking University Shenzhen Hospital and Southern Medical University Institutional Board.

### Cell culture

The normal human urinary tract epithelial cells SV-HUC-1 were obtained from ATCC (Manassas, VA, USA) and cultured in F-12K medium (Gibco). Four human bladder cancer cell lines (i.e. UM-UC-3, EJ, T24, RT4 and 5637) were obtained from the Foleibao Biotechnology Development (Shanghai, China). UM-UC-3, EJ and 5637 cell lines were routinely cultured in RPMI-1640 (Gibco, Grand Island, NY, USA), while T24 and RT4 cell lines was cultivated in DMEM (Gibco). All the media contained 10% heat-inactivated fetal bovine serum (HyClone, Logan, UT, USA). Cells were cultured and maintained at 37°C in an atmosphere of 5% CO_2_.

### Tissue microarray construction & immunohistochemistry (IHC)

Tissue microarray (TMA) was established from 155 formalin-fixed paraffin-embedded (FFPE) human bladder cancer tissues blocks according to the standard methods. HnRNP-L protein expression was confirmed using an immunoperoxidase method. The tissue array was deparaffinized, rehydrated, and inhibited endogenous peroxidase activities by 0.3% H_2_O_2_ for 30min. For antigen retrieval, the slides were boiled in sodium citrate buffer (10 mM, pH 6.0) in a pressure cooker for 7 min. Afterwards, nonspecific binding was blocked with 5% normal goat serum, and then incubated with primary antibody (monoclonal antibody hnRNP-L, 1:500, Abcam Inc., Cambridge, MA, USA). Sequentially tissue array was incubated with polyperoxidase-anti-mouse IgG (Zhongshan Biotech. China). Diaminobenzidine (DAB) was visualized as a chromogen substrate. The nucleus was counterstained with hematoxylin. hnRNP-L staining in nuclear was reckoned as detectable immunoreactions. To evaluate the consequences of hnRNP-L staining, the intensity and percentage of cells in cancerous and non-cancerous tissues were scored by two pathologists independently. The intensity of staining was determined in accordance with the following scale: 0 (no staining); 1 (weak staining, light yellow); 2 (moderate staining, yellowish brown) and 3 (strong staining, brown). Based on the percentage of positively stained tumor cells, the score of staining extent was denoted on 4point scale as follows: 0 (less than 5%); 1 (5 to 25%); 2 (25 to 50%); 3 (more than 50%). The final scores were then calculated according to score × proportion of positive tumor cells for hnRNP-L expression (range from 0 to 9). Tumor tissues with scores of 0–1 were recognized as low expression because approximately 90% of normal bladder mucosa expressed a low level of hnRNP-L with an IHC score of = < 1 in our preliminary study. Then we defined 0-1 as low expression and 2–9 as high expression. Immunohistochemistry (IHC) staining for Ki-67 (rabbit anti-Ki-67 polyclonal antibody, Abcam, 1:500) was conducted as previously described.

### Reverse transcriptase-polymerase chain reaction (PCR) and quantitative real-time PCR assays

Total RNAs was isolated from cultured cell lines or human bladder cancer specimens using Trizol procedure (Invitrogen Corporation, Carlsbad, CA, USA) according to the manufacturer's instructions. CDNAs were synthesized utilizing the PrimeScrip^TM^ RT reagent Kit (Takara) from 1μg of total RNA in 20 μl of reaction volume. Reaction mixture for qPCR executed on the ABI 7500 Fast Real Time PCR system (Applied Biosystems, Foster City, CA, USA) by using SYBR Premix Ex Taq^TM^ (Takara Clontech, Kyoto, Japan). GAPDH fragment was quantified as an internal control. The primer sequences were: Human hnRNP-L: 5′-TTGTGGCCCTGTCCAGAGAATT-3′ (forward) and 5′-GTTTGTGTAGTCCCAAGTATCCTG-3′ (reverse); human GAPDH: 5-ACAGTCAGCCGCATCTTCTT-3′ (forward) and 5′-GACAAGCTTCCCGTTCTCAG-3′ (reverse). Three independent replicates were executed for each experiment. The relative levels of each sample in comparison to control GAPDH was derived using the 2^-ΔΔ^Ct method.

### Western blot analysis

The tissue samples and cell lines total protein lysates was extracted using the radio immunoprecipitation assay (RIPA) buffer. Equal amounts of entire protein samples were separated by electrophoresis on SDS-polyacrylamide gel electrophoresis and electrotransferred from the gel to polyvinylidene fluoride (PVDF) membranes (Millipore Corporation, Billerica, MA, USA). The membranes were blocked with 5% fat-free milk or BSA, and then immunoblotted using the following primary antibodies: mouse anti-hnRNP-L was purchased from Abcam (Cambridge, MA, UK, ab6106) (1:1000), rabbit polyclonal antibody cleaved Caspase-3 (#9664), E-cadherin, Vimentin, Claudin-1, ZO-1, β-catenin, ZEB1, snail (#9782), phospho-ERK1/2 (Thr202/Tyr204) (#4370), phospho-JNK (Thr183/Tyr185) (#4668), phospho-P38 (Thr180/Tyr182) (#4511) (1:500) were from Cell Signaling Technology; rabbit polyclonal antibody caspase-3, caspase-6, caspase-9 were from Bioworld (St Louis Park, MN); rabbit polyclonal antibody Bcl-2, Bcl2-xL (Immunoway, USA) (1:500); mouse polyclonal antibody JNK (Proteintech Ltd., China) (1:500); mouse monoclonal anti-E2F1 (sc-251) was obtained from Santa Cruz Biotechnology, CA, USA. GAPDH (Proteintech Ltd., China) (1:1000) was utilised as an internal control. Finally, the membranes were incubated with the appropriate secondary antibodies (Boster Ltd., Wuhan, China) (1:5000). Signals were visualized by the enhanced chemiluminescence (ECL) detection system (Pierce Biotechnology, Rockford, IL, USA) acting in accordance with the manufacturer's instructions.

### Establishment of stably transfected cell lines

Lentivirus-mediated hnRNP-L overexpression and knockdown in UM-UC-3 and EJ cells were achieved according to the manufacturer's instruction (GeneCopoeia, Carlsbad, CA, USA). In briefly, cancer cells were infected by hnRNP-L shRNA (sense:5′-GCUUGGAUCAAUCUAAGAUTTdTdT-3′, Antisense: 5′-AUCUUAGAUUGAUCCAAGCTTdTdT-3′) lentivirus and recombinant hnRNP-L lentiviruses, respectively. The control groups were transducted with empty lentivirus vector which encode a green fluorescent protein (GFP) open reading frame (lentivirus-GFP). Infected cells were selected with puromycin. The infection efficiency was validated by Western blotting analyses.

### Cell proliferation assay

CCK-8 cell proliferation assay (CCK-8, Kit Dojindo, Kumamoto, Japan) was carried to measure the cell growth rate according to the manufacturer's instructions. Briefly, the UM-UC-3 and EJ cells at 1 × 10^3^ per well were placed in 96-well plates and cultured for 24 hours. Then, 10ul CCK-8 solutions were added and the absorbance values at 450nm on 1, 2, 3, 4 and 5 days were measured after 2 hours. Three independent replicates were measured per assay.

### Colony formation assay

Cells were seeded at a density of 5 × 10^2^ per well in six-well plates and cultured at 37°C for two weeks and then washed twice with PBS, fixed with 4% paraformaldehyde, stained with Giemsa. The number of colonies was photographed and counted using a microscope. Each assay was performed with three replicates.

### Cell cycle analysis

The effect of hnRNP-L on the cell cycle was confirmed by flow cytometry. In brief, cells were collected in ice-cold PBS and then fixed with 70% cold ethanol at 4°C overnight. The samples were removed from the ethanol with PBS and exposed to 125 U/ml RNaseA (Sigma. USA) for 30 min at 37°C. Subsequently, cells were stained with 50ug/mL propidium iodide (PI) (Keygentec. Nanjing, China) solution for 30 min in darkness and then thecell cyclee phases was determined by flow cytometry (FACSCalibur, Becton Dickinson). Each assay was replicated three times.

### *In vitro* apoptosis assay by flow cytometry

The impact of hnRNP-L on cell apoptosis was performed by using Annexin V-APC Apoptosis Detection Kit (Keygentec, Nanjing, China) according to the manufacturer's protocol. The apoptosis cells rate was determined by BD FACSC alibur system.

### Tumor xenograft *in vivo*

Female BALB/c-nu/nu athymic mice (4–5 weeks old) were purchased from Animal Center of Southern Medical University, were kept on specific pathogen-free conditions and bred in accordance with institutional guidelines. To evaluate the bladder cancer tumor growth *in vivo*, 5 × 10^6^ of EJ cells stably expressing hnRNP-L shRNA and negative control via lentiviral vector were separately injected subcutaneously and bilaterally inoculated into the athymic mice (10 mice per group) on the flank regions of legs. UM-UC-3 cells stably overexpression of hnRNP-L and negative control were also subcutaneously inoculated into the athymic mice (6 mice per group) Tumor volume was measured from two perpendicular axes and calculated using a formula: volume = (length × width^2^)/2. After 25 days, mice were euthanized using CO_2_ inhalation and tumors were harvested. Tumors were removed and weighed. Then primary tumors were fixed, paraffin-embedded and sectioned. Subsequently, these sections were observed under a microscope after haematoxylin and eosin (H&E) staining. All the procedures were approved by the Institution Animal Care and Use Committee of Southern Medical University.

### Cell wound healing assay

Cells (5 × 10^4^) were harvested with trypsinization, washed with PBS and planted on six-well culture seeds at a density of cells per/well in complete medium. After confluence, cells were scratched wounds with a sterile 10μl plastic pipette tips. Then, cells were washed twice with PBS and cultivated in serum-free culture medium. Wound closure and migration were monitored and photographed at 0 h, 24 h and 48 h under an inverted microscope. Cell motility was quantified by obtaining the distance between the advancing margins of cells in three randomly selected microscopic fields (×200) at each time point. For each well, three distinctive fields along the scratch were measured in triplicate.

### Transwell migration assay

Migration Corning (Costar, Corning, Cambridge, MA, USA) chambers (8.0 μm pore sizes) were rehydrated with serum-free RPMI-1640 for 2 h at 37°C. Cells were suspended in serum-free media and seeded in the upper compartment (5 × 10^4^, 200ml/well). Then 500 ul 10% FBS complete medium was added in the lower chamber. After incubation for 24 h, the cells in the lower coated membrane were fixed with methyl alcohol and stained with Giemsa (Boster Ltd., Wuhan, China). Lastly, the consequences were photographed under a light microscope in five randomly chosen fields visual fields (×200). Three duplicate wells were tested per assay.

### Statistical analysis

Statistical analyses were performed using the SPSS software (SPSS Standard version 16.0; SPSS Inc., Chicago, IL, USA). Results were characterized as mean ± SD or median. The differences were calculated utilizing *c*^2^ tests for categorical variables and ANOVA or Student's *t*-test for continuous variables. Kaplan–Meier survival curve was plotted from progression-free survival data and examined by the log-rank test. Hazard Ratio (HR) was decided using the Cox model. The *p* value < 0.05 was considered as significant.

### Abbreviations

HnRNP-L: heterogeneous nuclear ribonucleoprotein L; qRT-PCR: Quantitative real-time polymerase chain reaction; FACS: fluorescence-activated cell sorting; shRNA: short hairpin RNA; EMT: epithelial-to-mesenchymal transition; mRNA: messenger RNA; NC: negative control; MAPK: mitogen-activated protein kinase; ERK: extracellular signal-regulated kinase; JNK: c-Jun N-terminal kinase.

## SUPPLEMENTARY MATERIALS


