# Supplementary material for: HnRNP-L mediates bladder cancer progression by inhibiting apoptotic signaling and enhancing MAPK signaling pathways
Source: Oncotarget. 2017 Jan 11;8(8):13586–99. doi: 10.18632/oncotarget.14600 (PMC5355122; doi:10.18632/oncotarget.14600)
Supplement: Supplementary file 1 [file oncotarget-08-13586-s001.pdf]

HnRNP-L mediates bladder cancer progression by inhibiting apoptotic signaling and enhancing MAPK signaling pathways

Supplementary Materials

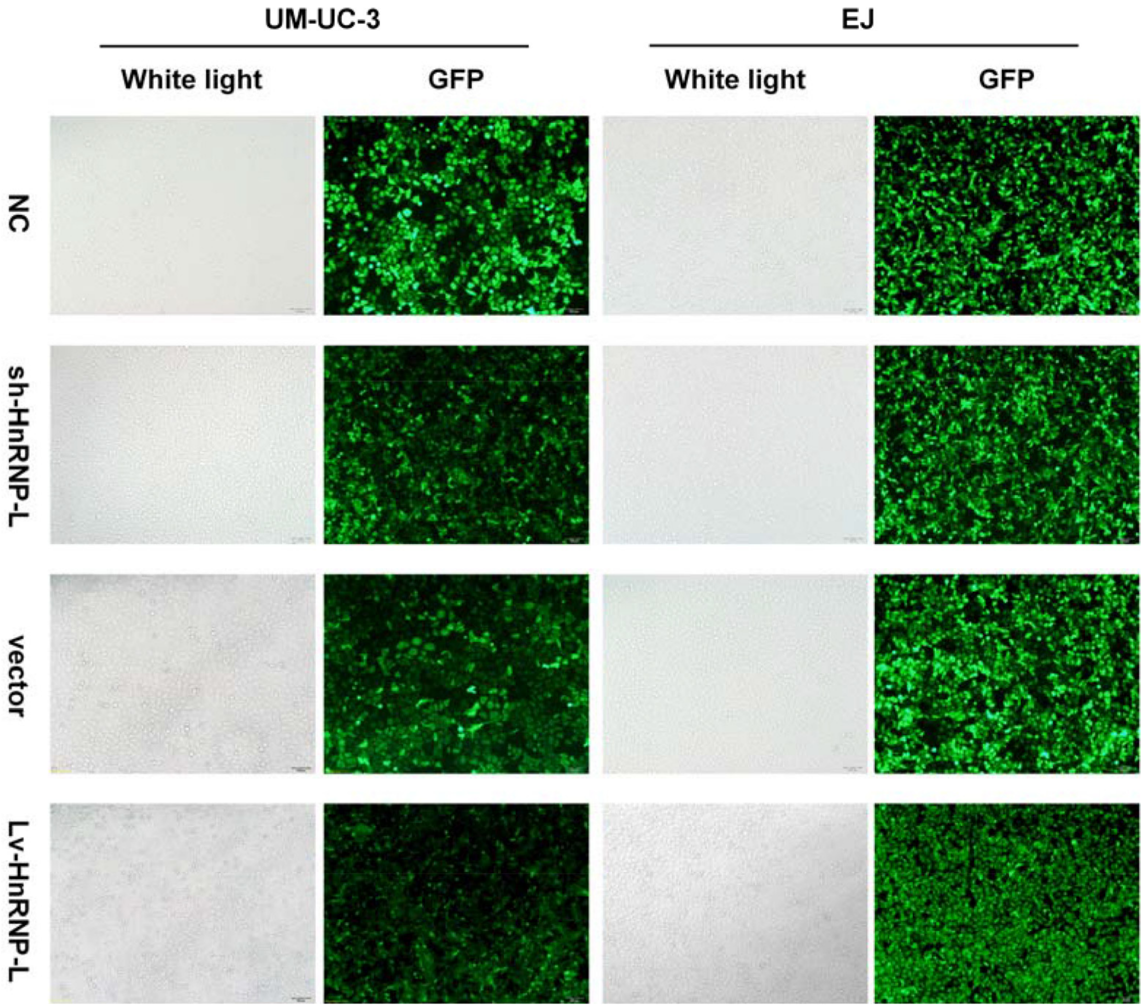

Supplementary Figure 1: The infection efficacy of lentivirus with GFP.

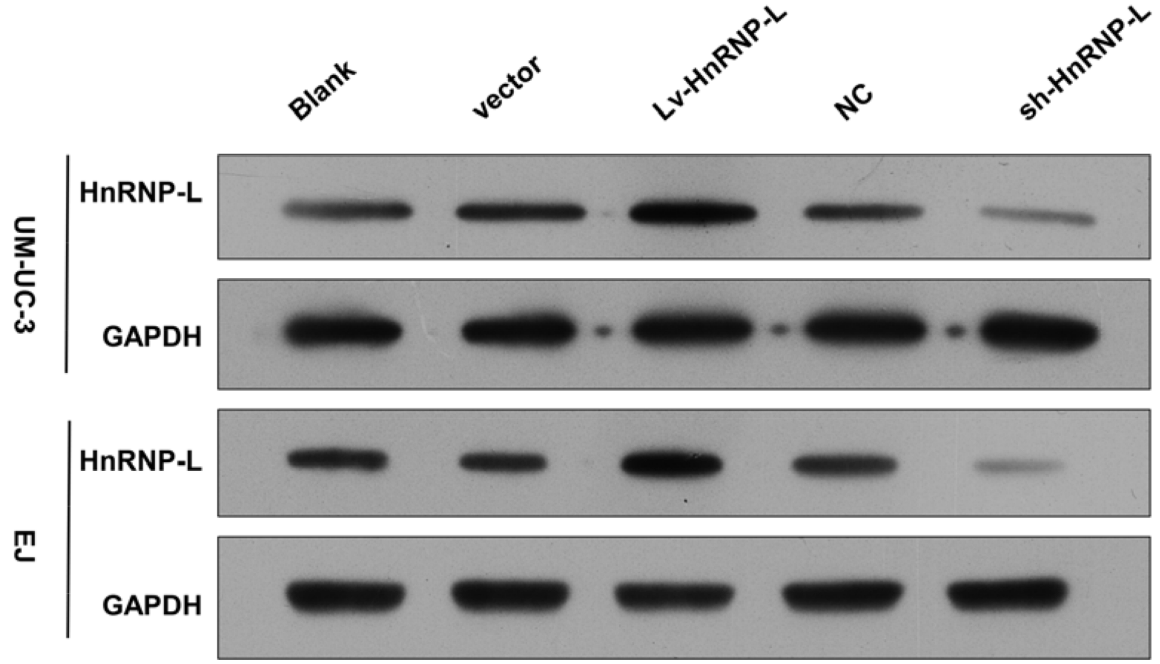

Supplementary Figure 2: Validation of the infection efficiency. Lentivirus-mediated knockdown and overexpression of HnRNP-L in UM-UC-3 and EJ cells were evaluated by Western blot for the protein level.
